# Clinical Research Primer for Medical Students: Behind the Curtain, a Framework on Peer Review for Trainees

**DOI:** 10.1055/a-2554-2357

**Published:** 2025-03-31

**Authors:** Taylor Niznik, Sherwin A. Tavakol, Tressie Stephens, Andrew M. Bauer, Ian F. Dunn, Christopher S. Graffeo

**Affiliations:** 1 Department of Neurosurgery, University of Oklahoma, Oklahoma City, Oklahoma, United States

**Keywords:** peer review, education, medical student, UME, GME

## Abstract

Academic scholarship is an increasingly emphasized component of undergraduate medical education (UME), in particular since the USMLE Step 1 examination transitioned to a pass/fail grading scheme in 2022. Peer review is a cornerstone of academic publishing, but essentially no formal training exists at the UME or graduate medical education levels to prepare trainees for participation in the process as authors or reviewers. This clinical research primer presents an introductory set of guidelines and pearls to empower trainee participation in the peer-review process as both authors and reviewers. We outline a systematic approach to manuscript evaluation and recommend a nonlinear strategy that begins with the Abstract and Methods, followed by Figures, Tables, and Results, concluding with the Discussion. This framework includes guidelines for constructing effective reviews, from initial summary and overall recommendations to specific, actionable comments. Participation in peer review can also advance trainees' scholarly development by exposing gaps in literature that inspire new research questions and developing their ability to anticipate and address potential reviewer critiques in their own manuscript preparation. While initial implementation requires close supervision from experienced mentors, this structured approach streamlines the peer-review learning process and provides substantial benefits for all participants in academic publishing, enhancing both mentorship relationships and scholarly development.

## Introduction

Academic scholarship is an increasingly emphasized component of undergraduate medical education (UME), in particular since the USMLE Step 1 examination transitioned to a pass/fail grading scheme in 2022. 1 Broadly, the infusion of new interest in research as a core component of UME curricula is poised to benefit students, educators, and patients alike; however, resources for UME students to develop their understanding of and familiarity with the core concepts of high-quality scholarship are lacking. 2 Competitive specialties such as neurosurgery and otolaryngology have lead the charge in developing novel, comprehensive initiatives that are tailored to the needs of medical students seeking to develop their research skills and resumes. 3 Although relatively novel, these initiatives have been preliminarily associated with higher levels of engagement among students aspiring to match in these specialties. 4 5 Peer review is a cornerstone of academic publishing, but essentially no formal training exists at the UME or graduate medical education (GME) levels to prepare trainees for participation in the process as authors or reviewers. This represents an important knowledge gap, given that integrating peer-review education into UME and GME curricula has the potential to inform students' perspectives on scholarship, as well as their practical skill sets for conducting and critiquing original research. 6 With these goals in mind, we sought to provide an introductory set of guidelines and pearls to empower trainee participation in the peer-review process as both authors and reviewers.

## Overview of the Peer-Review Process

Three key players comprise the peer-review dynamics—the editor, the author, and the reviewer. Although journal policies and protocols vary, these three archetypal roles are preserved across essentially all of academic publishing, as are the motivations and responsibilities inherent to each. The editor's primary goal is to curate and publish high-impact papers that enhance the journal's reputation and visibility. By selecting manuscripts that are likely to be read and cited, the editor not only boosts the journal's impact factor, but also ensures that the published content aligns with the journal's mission, enhancing long-term value and therefore retention of readership. To achieve this, editors depend on high-quality submissions from authors, and reliable, timely critiques from reviewers with salient expertise. These goals must be achieved while minimizing author frustrations and maintaining an efficient submission and review process that develops a reliable base of serial submissions and reviews.

By token, authors are driven by the need to publish their work, ideally in high-impact journals that will increase the visibility and citation potential of their work, thereby contributing to professional advancement, reputational development, and most importantly the more widespread dissemination of their ideas. Authors are typically motivated by a peer-review process that is simultaneously efficient and fair, and most authors publish predominantly in specialty-specific journals that have relatively high acceptance rates for similar submissions. Common sources of frustration that may decrease the potential for authors to preferentially submit to a given journal include protracted review processes with substantial delays at each phase, or capricious editorial decision-making, where good-faith efforts to meet reviewer recommendations do not result in acceptance for publication.

Finally, reviewers are the lynchpin in the system, ideally providing their most objective and unbiased perspective on the submitted work in order to facilitate the interests of the editor and author alike. Like editors, reviewers are volunteers who have donated their time and energy to the journal and its peer-review process; however, if the editor is like a curator, the reviewer is the critic, providing a detached source of insight and feedback to the editors that putatively helps maintain their standards of integrity and quality. Some journals blind reviewers to author identity in order to reduce the risk of bias, although this practice is far from universal, potentially opening the door to unconscious bias on the part of the reviewer. This is particularly problematic for niche topics, where the number of true topic experts other than the authors are individuals with whom they are in direct competition. The use of multiple reviewers and editorial discretion are helpful in that regard, but like many aspects of the peer review, certain intrinsic vulnerabilities simply cannot be fully circumvented, and the process thrives largely due to the carefully balanced counterpoint between the interests and incentives of the three key players.

## A Framework for Learning and Teaching Peer Review

**Fig. 1:**
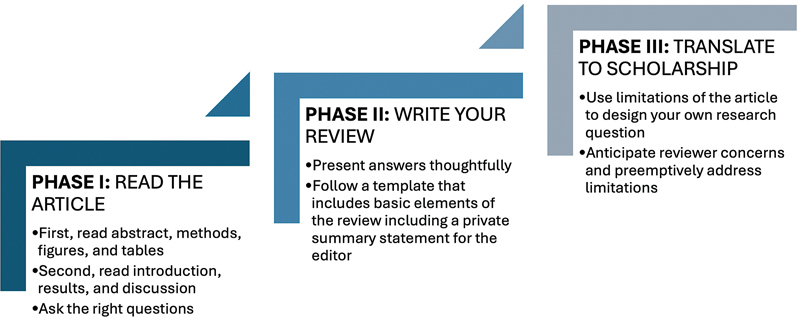
A framework for peer review as a trainee.

A structured approach to peer review allows students to build their skills systematically ( Fig. 1 ). The following framework provides an overview of the essential steps for reading, analyzing, and writing a peer review.

### Reading and Analyzing the Manuscript

When critically reading an article as a reviewer, we recommend adopting a nonlinear strategy in order to prioritize the highest-yield components of the manuscript, from the perspective of critical appraisal. This begins with the Abstract, which provides a comprehensive overview of the work, followed by the Methods. As the reviewer, you are also responsible for formulating a nuanced impression of whether the study design, data collection techniques, analytic strategies, and statistical testing were correctly selected, which is why a thorough assessment of the Methods should be prioritized. A critical assessment of the statistical methods is particularly important, as the validity of the study's conclusions often depends on whether the chosen analyses appropriately address the research questions. If you lack expertise in certain methodological areas (such as statistical analysis), it is prudent to communicate this limitation to the editor and potentially recommend additional expert review. Published data show that while peer review generally improves statistical reporting in manuscripts, many journals lack specialized statistical reviewers, highlighting the importance of acknowledging one's limitations in this area. 7 8 Once you have a clear understanding of the Methods, turn to the Figures, Tables, and Results, which represent the output of the Methods, and the objective content of the analysis. At this point in the review, you should have a clear sense for what was done, and ideally you should be formulating your own interpretation of the results to compare against the authors' own interpretation when you finally reach the Discussion.

One global concept to consider throughout your review is: “What core question is posed by this study, and was the study designed and executed in a way that addressed the question appropriately?” If not made clear by the Abstract, the study question should certainly be expressed in the Introduction, alongside an overview of the salient context and preceding literature on the topic. If the question is inadequately articulated, the study may have a more descriptive posture, or the writing itself may be an issue. If a question is well-articulated in the Introduction but inadequately addressed by the Methods, keep this in mind as you turn to the Discussion, and in particular look for a Limitations section, where the authors may have offered a justification for the unsatisfactory outcome of the study with regard to the question they posited.

More broadly, the Discussion should be considered the authors' opportunity to explain their results in context, to provide an interpretation of their findings, and to draw inferences or make recommendations on the basis of those interpretations. Consider whether the author's narrative is well supported by the data and results presented. Additionally, a literature search may be valuable to verify that the authors have included recent key publications and have not duplicated data published elsewhere. Pay particular attention to whether the conclusions are reasonable, balanced, and reflective of the study's findings. Finally, evaluate how the results are generalized from a small, focused manuscript, to the broader and more undifferentiated sphere of clinical practice.

Put another way, in addition to commenting on whether they have answered their study's question, the authors should be placing this information into a meaningful context and helping the reader decide whether the work will translate into a change in their own practice. High-quality manuscripts are consistent and transparent in how they negotiate these challenging questions, and although the Discussion is arguably the least useful component of a research project, a thoughtful and measured analysis of their work credits the authors with insight and understanding and enhances the potential influence of the publication.

### Writing the Review

Although we recommend taking brief notes throughout the reading process to inform your critical appraisal of the work, your formal role as a peer reviewer depends on the generation of the review itself, which is a sort of mini-manuscript unto itself. Your review will be read by the editor first, informing their decision to accept, reject, or request revisions from the authors; it will then be read by the authors, independent of the decision. Peer review depends on transparency as a core principle, and the authors receive the reviewer feedback both as a means to understand the decision, and an avenue for improving the work for revisions or resubmission to another journal. Correspondingly, your review should be clear, thoughtful, articulate, and specific, with an eye toward the author as a true peer you are attempting to help with their academic endeavors.

For novice reviewers in particular, a structured template will helpfully organize your review in a way that is helpful to the editor, the authors, and yourself—for example, should you be asked to review a future revision of the same submission ( Table 1 ). Many journals provide such structured review templates that address all parts of the process, and adopting these templates can facilitate reviews not only for the current submission but also for other journals or manuscripts in preparation. Begin your review with a concise paragraph that summarizes the article, including one sentence for each major section. Explicitly state the study design and setting, major methodological components, key results, and the author's interpretation of their findings. This summary allows you to succinctly capture the core content and serves as a quick reference for the editor and for yourself when reviewing future revisions, a process that likely will occur several months later. Following the summary, we recommend providing a brief 1- to 2-sentence critical overview in a separate paragraph that offers your overall evaluation of the manuscript for the authors, potentially to include a specific recommendation (e.g., “Accept without revisions,” “Minor revisions,” “Major revisions,” or “Reject”). Ideally, the editor should be able to infer your judgment and recommendations without reading any further into the review.

**Table 1:** Template for peer review

Review element	Example
One paragraph summary of the article with approximately one sentence per section	The authors report… Using a cohort study design… They observed… Based on these results, the author concluded…
Brief overall critique providing your summative review for the author and editor	Overall, the study is interesting, timely, and focused on a practical question with the potential to directly impact patient care. Notwithstanding, several methodological issues require attention at this time. Specific comments include the following:
List comments in an organized fashion, either by decreasing importance (preferred) or in chronological order with respect to the manuscript sections	(1) The main concern is regarding unadjusted selection bias in the overall study cohort…
Provide a private summary statement for the editor	A sufficiently novel case to merit publication, once SMRA issues addressed…

The final and most critical section of the written review is a series of specific, actionable comments for the authors to incorporate into their revisions, which are read and disseminated regardless of whether a resubmission offer is extended by the editor ( Table 2 ). Your comments should be presented in a clear and organized manner, either by following the manuscript's structure (e.g., Abstract, Introduction, Methods, etc.) or by listing the comments from most to least important. The latter is a more typical strategy as it highlights the biggest issues for both the editor's and the authors' reference; however, both are considered acceptable strategies. For most publications, it is conventional to provide at least 3 to 5 major comments. In rare cases, a truly excellent manuscript may require minimal commentary, save for your summary praise; however, more frequently, a larger number of points require enumeration. Regardless of the number of comments, anything you highlight needs to be specific and actionable, and your requested revisions need to be reasonable in scope, considering the project's nature, setting, and resources. Several minor comments may also be needed to address less impactful issues such as grammar, formatting, and tone, but these are ultimately less significant than major comments focusing on methodological issues, or errors in the analysis or interpretation of study data. Conclude your review with a private summary statement intended for the editor, offering your final assessment and any relevant recommendations, which will be submitted separately via the reviewer portal, so that they are not shared with the authors.

**Table 2:** Pearls for a successful peer review

Focus on the methods: great papers excel and poor ones falter upon examination of their methods, making this section an easy target for meaningful, objective critique
Consider the journal's focus: tailor your review to the journal's specific focus and audience
Provide specific recommendations: offer clear, actionable suggestions for improving the manuscript
Offer feasible recommendations: suggest practical and achievable suggestions for manuscript improvement
Organize your approach: structure your review systematically for clarity and coherence

### Translation to Scholarship

Participating in peer review not only provides insight regarding this critical component of academic volunteerism for possible future academicians, but students will also find that becoming facile with peer review will readily advance their own research in several ways.

One key lesson is how participating in peer review often exposes limitations or gaps in the literature, which in term may be used by the reviewer to formulate new research questions or develop complementary analyses (e.g., systemic reviews, meta-analyses, cohort study, narrative reviews, etc.). This analytical process of identifying research opportunities makes peer review an essential skill for trainees to develop, as it not only strengthens their ability to critically evaluate medical literature but also helps them contribute to advancing scientific knowledge. Journal clubs offer an excellent forum for practicing and refining these skills. Once students identify a potential research question, they should develop a detailed plan outlining the next steps for the proposed project and revisit the guiding questions of high-quality peer review.

In tandem, another important outcome of participating in peer review as a trainee is the enhanced ability to anticipate and preemptively address potential reviewer comments during your own study design and manuscript drafting processes. Once you are able to “put your reviewer hat on,” your eyes will be opened to flaws in your work, and you will be able to ask yourself from a more well-informed perspective whether you have acknowledged and justified these shortcomings in a way that you would consider acceptable as a reviewer. It may be helpful to involve coauthors as informal reviewers to provide additional critiques in this regard; similarly, when you are a middle author on a paper, being able to point out possible reviewer critiques of a late draft prior to submission may likely provide critical insight for the lead and senior authors. As you finalize your own manuscripts, consider how they may serve the needs of the editors and readers by providing interesting, impactful, and robust contributions to the literature, resting on sound methods and plausible interpretations. As an author, your responsibility is to ensure that any remaining shortcomings are transparent and justified, and that they do not substantially comprise the integrity or certainty of the work prior to submission.

## Conclusion

Peer review is a valuable skill for medical trainees aspiring to careers in academic medicine. By taking a systematic approach to critically reading and reviewing submitted manuscripts, as well as providing constructive feedback in the form of a written review, students will not only improve their own skills as researchers, but they will also gain deeper insights into the process of academic publishing. Initially, these contributions require close supervision from a mentoring attending with robust experience in the peer-review process; however, we anticipate that the processes we have outlined will streamline these important relationships on behalf of both the mentor and the mentee, with substantial downstream benefits for all participants and their scholarly work.
